# Activating Childcare Environments for All Children: the Importance of Children’s Individual Needs

**DOI:** 10.3390/ijerph15071400

**Published:** 2018-07-03

**Authors:** Jessica S. Gubbels, Dave H. H. Van Kann, Greet Cardon, Stef P. J. Kremers

**Affiliations:** 1 Department of Health Promotion, NUTRIM School of Nutrition and Translational Research in Metabolism, Faculty of Health, Medicine and Life Sciences, Maastricht University, P.O. Box 616, 6200 MD Maastricht, The Netherlands; s.kremers@maastrichtuniversity.nl; 2 Research Group Move to Be, School of Sport Studies, Fontys University of Applied Sciences, P.O. Box 347, 5600 AH Eindhoven, The Netherlands; d.vankann@fontys.nl; 3 Department of Movement and Sports Sciences, Ghent University, C. Heymanslaan 10, 9000 Ghent, Belgium; greet.cardon@ugent.be

**Keywords:** childcare, sedentary behavior, physical activity, toddlers, preschoolers, interaction, moderation, temperament, physical environment, natural elements

## Abstract

Characteristics of the physical childcare environment are associated with children’s sedentary behavior (SB) and physical activity (PA) levels. This study examines whether these associations are moderated by child characteristics. A total of 152 1- to 3-year-old children from 22 Dutch childcare centers participated in the study. Trained research assistants observed the physical childcare environment, using the Environment and Policy Assessment Observation (EPAO) protocol. Child characteristics (age, gender, temperament and weight status) were assessed using parental questionnaires. Child SB and PA was assessed using Actigraph GT3X+ accelerometers. Linear regression analyses including interaction terms were used to examine moderation of associations between the childcare environment and child SB and PA. Natural elements and portable outdoor equipment were associated with less SB and more PA. In addition, older children, boys and heavier children were less sedentary and more active, while more use of childcare and an anxious temperament were associated with more SB. There were various interactions between environmental factors and child characteristics. Specific physical elements (e.g., natural elements) were especially beneficial for vulnerable children (i.e., anxious, overactive, depressive/withdrawn, overweight). The current study shows the importance of the physical childcare environment in lowering SB and promoting PA in very young children in general, and vulnerable children specifically. Moderation by child characteristics shows the urgency of shaping childcare centers that promote PA in all children, increasing equity in PA promotion in childcare.

## 1. Introduction

Physical activity (PA) during childhood can have positive effects on a broad spectrum of health and developmental outcomes. This includes effects on adiposity, cardiovascular health, bone health, motor skills, mental health, self-esteem and academic performance [1,2]. Moreover, PA habits are rooted at a young age and often maintained throughout life [3,4]. Targeting PA in early childhood is therefore essential.

The preschool a child attends is significantly associated with the child’s total PA [5]. The childcare or preschool setting thus offers an opportunity for promoting PA and preventing sedentary behavior (SB) among young children [6]. However, studies across countries show that children are predominantly sedentary at childcare or preschool (e.g., [7,8,9]). A recent review showed that even during outdoor play sessions at childcare, children are mostly sedentary [10]. Moreover, children appear to be less active during childcare hours compared to home [11].

A review on correlates of SB and PA in early childhood education and care services, concluded that the physical environment has the most significant influence [12]. Especially play opportunities and size of the outdoor play space seem consistently associated with SB and PA levels [12]. Additional physical environmental features linked to less SB and/or increased PA include natural elements [13], playgrounds markings [14,15], ground surface [16,17], and specific types of play equipment [8,9,15,17,18].

In addition to environmental predictors of SB and PA, there is a large evidence base showing higher activity levels in boys and older children (e.g., [12,19]). Tonge and colleagues have speculated that these differences might be caused by childcare environments that are more suitable for boys and older children [12]. This suggests that children are stimulated differentially by the environment. In line with this, ecological models propose that the influence of the environment on behavior be moderated by the characteristics of the child [20,21]. Several studies in fact have shown such moderation by age and gender [21]. Age moderated the effect of playground markings, having stronger positive effects on younger children [14]. Older children were found to increase their vigorous activity in response to a playground intervention, while younger children mainly increased their moderate activity [22]. Regarding gender, boys’ activity levels were positively associated with natural ground coverings [11] and the overall quality of the outdoor environment [13], and negatively with soft ground surfaces [18]. In girls, these associations were absent [13,16]. However, girls profited more from increasing the available play space [23]. 

To date, moderation analyses of childcare influences on SB and PA are limited to the demographic characteristics described above (age and gender). Research in settings other than childcare (e.g., schools, home) indicates child weight status as a moderator of environmental influences on children’s SB and PA (e.g., [24,25]). Similarly, temperament (the combination of mental, physical and emotional traits of a person) has been theorized as a potential moderator of environmental influences [20,26]. These additional potential moderators should also be examined at childcare, as ignoring such interactions could lead to suboptimal intervention effects, wasting valuable time and money [21]. The current study therefore aims to examine the combined and interacting influence of child characteristics and the physical childcare environment, on children’s SB and PA levels at childcare. 

## 2. Materials and Methods

### 2.1. Respondents and Procedure

A total of 174 randomly selected childcare centers in the southern provinces of The Netherlands (Noord-Brabant and Limburg) were approached by email and/or telephone to participate in the study. Only centers offering full-day care were included. Twenty-three centers (13.2%) agreed to participate. All parents of the children aged 1 to 4 years old from these centers were invited by letter or email to participate in the study. In addition, we used face-to-face on-site recruitment at drop-off and pick-up times. Parents of 218 children agreed to participate and provided written informed consent. Six children were excluded because they could not walk independently (*N* = 1) or were indicated as too young for the study by their parents (*N* = 5), leaving 212 children for the current study. The data collection period ranged from November 2014 to January 2016. The study was exempted from full medical ethical review and approved by the Maastricht University Medical Centre+ medical ethics committee, under the Dutch law Medical Research with Human subjects (WMO).

### 2.2. Measures

#### 2.2.1. Physical Childcare Environment

At each childcare center, the physical environment regarding SB and PA was assessed by a trained research assistant. A standardized observation protocol was applied, based on the updated Environment and Policy and Assessment Observation (EPAO; [27]). Portable and fixed play equipment were separately rated for indoors or outdoors, as either present or not. The following portable equipment was assessed: balls, portable climbing structures, floor play equipment (e.g., tumbling mats), jumping play equipment (e.g., bouncing balls), push/pull toys (e.g., doll wagon), riding toys (e.g., tricycles), rocking or twisting toys (e.g., rocking horse), sand/water tables, sand/water play toys (e.g., scoops), portable slides, small portable pools, portable tunnels and other portable equipment. The number of portable equipment types was summed separately for indoor and outdoor. The fixed equipment assessed were balancing surfaces (e.g., balance beams), basketball hoop or soccer goal, fixed climbing structures, merry-go-round, fixed place to play with water, sandbox, see-saw, fixed slides, swinging equipment (e.g., swings), tricycle track or paved areas, fixed tunnels, benches, picnic tables, small stage or raised deck, play house, floor markings (e.g., colors, tracks), and other fixed equipment. In line with the portable equipment, separate indoor and outdoor sum scores were calculated for fixed equipment. 

Indoor areas for active play were assessed using the following question, completed by the observer: “When children are inside, where do they participate in physically active play (gross motor activities such as running, jumping, hopping, tumbling)?”. Answering options were “the classroom”, “gym or multipurpose room”, “hallway”, “other”, and “no room inside for these activities”. The number of indoor areas was then summed. The number of outdoor play areas was assessed by asking the open-ended question “How many play spaces are there outdoors for preschool children?”. 

Natural elements were assessed by observing “Which of the following does the center have outside where children are allowed to play?”. Answering options were “large trees (2.5 m or taller)”, “small trees (less than 2.5 m tall)”, “trees that children can climb”, “shrubs”, “flowering plants”, “variation in ground (hills, mounds)”, “grass”, “rocks large enough to climb”, “a hill for rolling down or climbing up”. A sum score of all the types of natural elements that were present was calculated. 

Finally, the observer measured the size of the outdoor play area and any obstacles in meters, using measurement tape. Total free play space in square meters was calculated from this. In addition, the number of children present was recorded. Total free play space was divided by the number of children to obtain free space per child (in m^2^).

#### 2.2.2. Children’s SB and PA

Children’s SB and PA level was objectively measured using an accelerometer (Actigraph GT3X+, 30 Hz, Actigraph, Pensacola, FL, USA). The epoch was set to 10 s. Children wore the accelerometer for seven consecutive days on the right hip during waking hours, except during swimming, showering and other water activities. Children did not wear the accelerometer during sleeping. Wear time validation criteria by Troiano (2007) were used [28]. Daily sum variables of the accelerometers were used. To be included in the analyses, minimal wear time per day was 360 min. Based on a review of available cut points [29], uniaxial cut points by Pate and colleagues for preschoolers [30] were considered most appropriate and were applied to extract the time spent sedentary and in moderate to vigorous PA (MVPA). Time spent in these categories was divided by total wearing time to calculate percentage of wearing time spent sedentary and in MVPA. In addition, average counts per minute (CPM) based on vector magnitude were extracted. 

During the measurement week, parents indicated in a diary on which days their child had attended childcare. Based on these diaries, childcare days were selected from the accelerometer data. The accelerometer data used in the current study thus reflect the SB and PA on days on which children attended childcare (including the remaining time spent at home on such a day, typically a few hours in the morning and evening, as only full-day care was included). All children with at least one valid childcare day were included. For children providing valid data for more than one childcare day, the average over all childcare days was calculated. The number of valid days per child ranged from 1 to 7 days. Season was derived from the first valid measurement day for each child. For the analyses, autumn was used as a reference category.

#### 2.2.3. Children’s Background Characteristics

Child gender and age (in months), as well as average usage of childcare (days per week), were assessed using an online parental questionnaire. In addition, children’s temperament was assessed in this questionnaire, using the Child Behavior Check List for toddlers (CBCL/2-3; [31]). The subscales assessed reflected an oppositional (17 items, e.g., “My child is stubborn”), withdrawn/depressive (10 items, e.g., “My child doesn’t answer when others talk to him/her”), anxious (10 items, e.g., “My child is easily upset by new people or situations”), or overactive (5 items, e.g., “My child cannot sit still”) temperament. For each of the 42 items, parents could indicate whether the items were “not true” (1); “somewhat or sometimes true” (2); or “very true or often true” (3) for the child. For each of the four scales, an average of the items was calculated. In addition to children’s characteristics, the questionnaire assessed both parents’ educational level. Educational level was recoded into low and high, using medium as the reference category. 

Trained research assistants measured children’s height and weight at the child-care center. Children wore light clothing, no shoes, and in case the child wore a diaper, this was changed before the measurement. Height and weight were used to calculate body mass index (BMI), which was converted to BMI z-scores, reflecting the number of standard deviations the child differed from the age- and gender-specific mean of the national reference population [32].

### 2.3. Statistical Analyses

Accelerometer data were available for 196 children (92.5%), parental questionnaires were available for 167 children (78.8%), and childcare observations were available for 196 children (92.5%). For 152 children (71.7%) from 22 childcare centers (95.7%), all three measures were available. 

All analyses were conducted using IBM SPSS Statistics for Windows, version 24.0 (IBM corp., Armonk, NY, USA). *p*-values < 0.05 were considered statistically significant. Descriptive statistics were used to examine all variables included in the study. Multiple multivariate linear regression analyses were conducted to examine the associations between the physical childcare environmental factors and child characteristics on the one hand, and children’s percentage SB and PA (average CPM and percentage MVPA) on the other hand, adjusted for season and parental educational level. In a second set of regression analyses, interactions between each of the physical environment and child characteristics were added to the models with child characteristics and environmental factors, to examine interactions. In case of a significant interaction term, the regression analyses examining the main effects were stratified based on the child characteristic involved.

## 3. Results

Table 1 shows the descriptive statistics of children’s characteristics, SB and PA. Average childcare use was 2 days a week. The children in the study had a positive average BMI *z*-score (0.38), indicating that they were heavier than the reference population. Children scored low on the temperament scales on average, with the highest averages for overactive and oppositional temperaments. Children’s activity level was mainly sedentary (82% of the time), while they were moderate to vigorously active for less than one hour a day (8%). Most accelerometer measurements took place in autumn (52.6%). 

Table 2 shows the physical environmental features. The 22 included childcare centers encompassed 28 different groups (on a physically different location; groups using the same location but on a different weekday were merged). Outdoor fixed equipment was most present (6.4 types on average); the other categories were much less present. There was an average of four natural elements on the playgrounds, although there were also centers that did not have any natural elements. There was a very large variation in the outdoor free surface.

### 3.1. Association of Physical Environment and Child Characteristics with PA

Table 3 shows the associations of the physical childcare environment and children’s characteristics with children’s SB and PA levels. Age was significantly associated with less SB and increased PA (higher CPM, higher percentage of MVPA). An anxious temperament was associated with more SB as well as decreased PA across outcomes. Higher childcare attendance was associated with increased SB during childcare days, while child’s BMI *z*-score was negatively associated with SB. Regarding the physical childcare environment, natural elements were consistently significantly and positively associated with less SB and increased PA across the outcomes. Outdoor portable equipment was associated with increased MVPA.

### 3.2. Interaction Physical Environment with Child Characteristics

Various child characteristics moderated the association between the physical childcare environment and children’s PA (interactions *p* < 0.05). Natural elements were significantly and positively associated with average PA (CPM) in anxious children (β = 0.37, *p* = 0.047), while this was not the case for children with low anxiety scores (*p* > 0.05). In addition, natural elements showed a negative association with sedentary time of 3-year-olds (β = −0.53, *p* = 0.047), but not of 1- and 2-year-olds (*p*’s > 0.05). Portable indoor play equipment showed a negative association with sedentary time in overactive children (β = −0.28, *p* = 0.022), but not in non-overactive children (*p* > 0.05). 

Outdoor play space (m^2^ per child) was significantly associated with all outcome measures for children who were overweight: it was negatively associated with sedentary time (β = −0.72, *p* = 0.026), while it was positively associated with average PA (CPM; β = 0.74, *p* = 0.009) and time spent in MVPA (β = 0.79, *p* = 0.020) overweight children. None of these associations were present in normal weight children (all associations *p* > 0.05). 

The number of outdoor play spaces was negatively associated with sedentary time (β = −0.34, *p* = 0.047), and positively with time in MVPA (β = 0.42, *p* = 0.016) in children scoring high on the depressive/withdrawn temperament scale. These associations were not present in non-depressive/withdrawn children (both *p* > 0.05). The number of outdoor play spaces was further positively associated with MVPA of 3-year-olds (β = 0.38, *p* = 0.035), but not of 1- and 2-year-olds (*p* > 0.05). 

## 4. Discussion

The current study examined the interacting influence of the physical childcare environment and child characteristics, on children’s SB and PA levels. Natural elements and outdoor portable equipment were significant positive environmental predictors of children’s activity levels. In addition, older children, boys and heavier children were less sedentary and more active, while more use of childcare and an anxious temperament were associated with more SB. Furthermore, there were various significant interactions between environmental factors and child characteristics. 

In line with previous studies (e.g., [12,19]), we found higher activity levels in boys and older children. In addition, childcare attendance was positively associated with SB. This could indicate inactivity as a potential mediator in the reported dose-response relationship between childcare use and overweight (e.g., [33]). Boredom might play a role in explaining why children become more sedentary with increasing childcare use: PA opportunities often become less challenging for children attending childcare multiple days a week [34]. An alternative explanation might be found in meso-system inconsistencies between home and the childcare setting: child outcomes seem to be negatively influenced by such inconsistencies between settings [21]. More days at childcare, means more frequent inconsistency, and as such might result in more SB. More research would be needed to confirm these hypotheses. Regarding temperament, an anxious temperament was associated with increased SB and decreased PA. In line with this, childcare staff indicate temperament as an important predictor of PA at childcare [35].

Presence of natural elements was consistently associated with less SB and higher PA levels, across activity outcomes. This is in line with research by Boldemann et al. [13], who reported a positive association of step count in preschoolers, with having trees, shrubbery and broken ground. According to Bjørgen (2016), natural environments offer more PA opportunities compared to non-natural outdoor environments [36]. Natural environments evoke play and fun, from which PA and other positive health effects will follow effortlessly [37]. However, there are studies reporting a negative association of vegetation on the preschool playground with PA [38], as well as studies reporting no association between vegetation and SB or PA (e.g., [16]). These conflicting findings can be caused by differences in the type of natural elements assessed: functional natural elements (e.g., grass, hills, natural elements to climb on) versus aesthetic natural elements (e.g., plants, high trees). Functional natural elements are expected to contribute to increased PA, while aesthetic elements might obstruct PA and evoke SB [39]. Additional research into these different functionalities of natural elements is needed to design future playgrounds optimally. Alternatively, the conflicting findings might be related to the differences in samples between the studies, as the effects of natural elements seem to be moderated by child characteristics. Natural elements were particularly beneficial for children with an anxious temperament and older children in the current study. Especially for anxious children this is very important, as they exhibited more SB and lower levels of PA in general. Nature might be able to pull them out of their sedition, but additionally have positive effects on their anxiety as well: contact with nature has been found to be beneficial for mental health in general, and anxiety specifically (e.g., [40]). 

Several non-natural elements were associated with SB and PA as well. It is important to note that although SB and PA were assessed throughout the whole day (indoor and outdoor), only *outdoor* childcare characteristics were associated with SB and PA levels. This is in line with previous research showing the importance of activity opportunities in the outdoor environment [12] and underlines previous studies in concluding that increasing outdoor time is essential (e.g., [41]). Weather conditions might be crucial in this respect, as bad weather is one of the major barriers for outdoor play mentioned by staff [42]. Outdoor portable play equipment was associated with increased MVPA in the current study. The number of available outdoor play spaces was positively associated with activity levels of older children and children with a depressive/withdrawn temperament. These children might benefit from the variation in play environments, preventing boredom. Some environmental features were associated with PA in 3-year-olds, but not in younger children. This seems logical, as the younger children are still in the very beginning of their gross motor development and thus not using equipment yet. Interestingly, outdoor play space (m^2^ per child) was positively associated with PA across outcomes for children who were overweight, but not for normal weight children. This positive association is in line with previous studies [13,17,18,43]; it is, however, unclear why we found this association only for overweight children. As we have no previous research to compare these moderating effects with, more research into moderation of environmental effects by child characteristics is urgently needed [21]. 

The fact that various child characteristics interact with the effect of the childcare environment, further indicates that different children need different opportunities to be active [11,21], and that our current arrangement of childcare centers is more suitable for some children than for others [12]. Physical childcare environments should be designed to provide opportunities to be active for all children [12]. This could potentially increase the effectiveness of PA promoting interventions, as well as help to address the disparity observed in health-related behaviors between children [21,44]. The physical environment showed to play a crucial role in providing PA opportunities for vulnerable children specifically: most interactions in the current study (except for those with age) pointed in the direction that specific physical environmental features have the largest positive effects on vulnerable children (those with anxious, overactive or depressive/withdrawn temperaments, and those who are overweight). In line with this, a large cross-European study on PA in adults showed that PA promoting physical environmental features are beneficial for everyone, but in some cases especially beneficial for vulnerable populations [45]. 

In addition to adjusting the physical environment, childcare supervisors can be made aware of the different responses of children to the environment and can be educated on how PA can be promoted in all children, with specific attention for vulnerable children [16,21]. This also indicates that some of the deeply rooted beliefs of childcare staff that some children are “naturally less active” [40] need to be addressed. PA promoting interventions should guide childcare staff (social environment) in supporting children in utilizing the activity opportunities offered (physical environment). Childcare staff, in turn, needs the support and guidance of a clear PA policy (political environment). Moreover, on a more distal level, city planners and politicians need to take their responsibility and back these efforts up by enabling environments that promote PA. In this regard, intersectoral collaboration is key [46]. Thus, comprehensive, multicomponent and multilevel approaches, focusing on environment, staff and policy, seem promising, from both a practical [47] and a theoretical [21,48] viewpoint. Taking things another step further, it seems that the most successful approaches include parents as well [49], aligning home and childcare within the bigger system of the child [21]. 

The current study has several strengths and limitations that need to be acknowledged. A strength of the current study is the use of objective measures for SB and PA (through accelerometers) as well as for the indoor and outdoor physical childcare environment (using a validated observation protocol by trained staff). However, accelerometer data regarded the whole day, including time spent outside the childcare center, possibly diluting potential effects. Choices regarding accelerometer epoch, cut points and wear time, among others, might have affected the results of the accelerometers. In addition, child characteristics were parent-reported. Furthermore, the sample size was limited, impeding correction for the multi-level structure of the data. In addition, we had no information on childcare centers and children that did not participate in the study, and therefore cannot rule out selection bias. This might limit the generalizability of our findings. Furthermore, health status was not assessed in the current study, but might have influenced the results. Infectious diseases often spread through childcare (e.g., [50]), which in turn can influence infected children’s PA levels. In addition, the Dutch childcare system was in a transitional phase during the data collection, hampering recruitment (e.g., because of center bankruptcies). Moreover, the data were cross-sectional. The main associations were, however, in line with previous research in the childcare setting, including intervention studies. Longitudinal studies and intervention studies addressing moderating effects of child characteristics will be needed to examine the interactions found in the current study further. 

## 5. Conclusions

The current study showed the importance of especially natural elements, but also of portable play equipment, in promoting very young children’s PA and preventing SB at childcare. In addition, moderation by several child characteristics showed the potential equity effect of improving the physical environment at childcare, shaping childcare centers that promote PA in all children, with specific attention for vulnerable groups. Children are not naturally inactive; they just have different needs when it comes to PA. It is our duty to inspire all children to profit from the benefits PA has to offer. 

## Figures and Tables

**Table 1:** Descriptive statistics of children’s characteristics and PA and measurement season (*N* = 152).

Variable			N (%) ^a^	Mean (SD)
**Gender**	Boy		72 (47.7%)	
	Girl		79 (52.3%)	
**Age (months)**				34.14 (8.97)
**Childcare use (number of days)**				2.01 (0.78)
**BMI *z*-score**				0.38 (0.96)
**Temperament ^b^**	Depressive/withdrawn			1.08 (0.15)
	Overactive			1.53 (0.40)
	Oppositional			1.47 (0.36)
	Anxious			1.34 (0.32)
**Physical activity ^c^**	Average counts/minute (vector magnitude)			1080.09 (277.46)
	Sedentary time	Minutes		510.31 (63.56)
		Percentage		82.35 (5.83)
	MVPA	Minutes		53.74 (20.66)
		Percentage		8.35 (3.70)
**Season**	Winter		48 (31.6%)	
	Spring		18 (11.8%)	
	Summer		6 (3.9%)	
	Autumn		80 (52.6%)	

Notes: BMI: body mass index, MVPA: moderate to vigorous activity, SD: standard deviation. ^a^ N’s deviate from total sample size due to missing values. Valid percentages are presented. ^b^ Assessed by the Child Behaviour Check List [31], scale 1–3. ^c^ Assessed by Actigraph GT3X+ accelerometer.

**Table 2:** Descriptive statistics of physical environmental features.

Environmental Feature	Range (min-max)	Mean (SD)
Indoor fixed equipment ^a^	0–4	1.9 (1.1)
Indoor portable equipment ^a^	0–7	1.8 (1.4)
Outdoor fixed equipment ^a^	1–10	6.4 (2.7)
Outdoor portable equipment ^a^	0–6	1.1 (1.7)
Natural elements ^a^	0–9	4.0 (2.1)
Number of play areas indoor	1–3	1.8 (0.6)
Number of play areas outdoor	1–3	1.4 (0.7)
Outdoor play area free surface (m^2^ per child)	6.2–221.7	42.9 (45.6)

Notes: Features of 28 different childcare groups from 22 childcare centers. SD: standard deviation. ^a^ Number of types.

**Table 3:** Backward multivariate regression analyses of the association between the physical childcare environment, children’s characteristics, and children’s PA levels (*N* = 152).

Independent Variables	Standardized Regression Coefficient (β) ^a^		
	Average Activity Level (CPM)	% MVPA	% Sedentary
Child gender (1 = male, 2 = female)	−0.26 **	-	-
Child age (months)	0.37 ***	0.35 ***	−0.40 ***
Child BMI *z*-score	-	-	−0.17 *
Childcare attendance (number of days)	-	-	0.19 *
Child anxious temperament	−0.21 **	−0.18 *	0.19 **
Outdoor portable equipment (number of types)	-	0.17 *	-
Natural elements (number of types)	0.21 **	0.27 **	−0.31 ***

Notes: CPM: counts per minute, MVPA: moderate to vigorous physical activity; %: percentage of total wearing time. * *p* < 0.05, ** *p*< 0.01, *** *p* < 0.001. ^a^ Variables excluded from the final models because they were non-significant for any of the outcomes: child oppositional, withdrawn/depressed and overactive behavior; number of play areas indoor and outdoor; Outdoor play area surface; Indoor fixed and portable equipment; outdoor fixed equipment; measurement season and parental educational level.
